# *In Silico* Evaluation of the Impacts of Quorum Sensing Inhibition (QSI) on Strain Competition and Development of QSI Resistance

**DOI:** 10.1038/srep35136

**Published:** 2016-10-13

**Authors:** Guopeng Wei, Chieh Lo, Connor Walsh, N. Luisa Hiller, Radu Marculescu

**Affiliations:** 1 Electrical and Computer Engineering, Carnegie Mellon University, Pittsburgh, PA, USA; 2 Biological Sciences, Carnegie Mellon University, Pittsburgh, PA, USA

## Abstract

As understanding of bacterial regulatory systems and pathogenesis continues to increase, QSI has been a major focus of research. However, recent studies have shown that mechanisms of resistance to quorum sensing (QS) inhibitors (QSIs) exist, calling into question their clinical value. We propose a computational framework that considers bacteria genotypes relative to QS genes and QS-regulated products including private, quasi-public, and public goods according to their impacts on bacterial fitness. Our results show (1) QSI resistance spreads when QS positively regulates the expression of private or quasi-public goods. (2) Resistance to drugs targeting secreted compounds downstream of QS for a mix of private, public, and quasi-public goods also spreads. (3) Changing the micro-environment during treatment with QSIs may decrease the spread of resistance. At fundamental-level, our simulation framework allows us to directly quantify cell-cell interactions and biofilm dynamics. Practically, the model provides a valuable tool for the study of QSI-based therapies, and the simulations reveal experimental paths that may guide QSI-based therapies in a manner that avoids or decreases the spread of QSI resistance.

While the era of antibiotics marks a cornerstone of modern medicine, it has, likewise, triggered the rise of virtually untreatable multidrug-resistant bacteria^1^,^2^. As new drug-resistant bacterial strains, such as carbapenem-resistant Enterobacteriaceae (CRE), continue to appear and spread, health officials are raising concern over the future efficacy of traditional antibiotics^3^. In response, substantial research efforts have shifted focus toward innovative targeted drug development strategies including anti-virulence therapy targeting cellular functions essential for pathogenesis within the human host rather than cellular vitality^4^.

Quorum sensing (QS) is a mechanism used by many bacteria to synchronize their collective behavior when reaching a sufficient high cell density^5^. In this paper, we consider the *LasI*/*R* QS system, which belongs to the *LusI*/*R* family of Gram-negative QS system. Specifically, members of the *LuxI* family produce *acyl homoserine lactones* (AHL) of varying acyl chain length that function as a signal. The signaling molecules bind to the receptors and activate the transcription regulator (*LuxR* homologs) in a form of *LasR* − *AHL* complex. This complex then leads to the transcription of a plurality of genes that are directly involved in bacteria collective behaviors^6^.

QS inhibitors (QSIs) aim at disabling the QS molecular signaling machinery within a bacterial pathogen, effectively rendering cells incapable of sensing the neighboring cell and consequently modifying the regulation of genes^7^. As a consequence, QSI modifies the regulation of genes such as biofilm formation, the production of secondary metabolites, and the expression of disease-causing virulence factors^8^,^9^,^10^,^11^.

Despite the preliminary success of QSIs (see Supplementary Note 1), there remain fundamental issues that may constrain their potential clinical merit. Principally, QS inhibition, since its inception, has been argued to be an “evolution-proof” therapy insofar that it precludes direct pressures on cellular fitness and thereby obviates the explicit selection of drug resistant genotypes^4^,^8^,^12^,^13^,^14^. Unfortunately, the validity of this claim has failed to hold true in light of recent accounts of QSI resistant strains found both in clinical and laboratory settings^15^,^16^,^17^. The major issue is that while the QS inhibition does not directly kill bacteria (bactericidal effect) or stop bacteria from growing (bacteriostatic effect) like conventional broad-spectrum antibiotics— it does, however, alter the behavior of targeted pathogens by modifying the expression levels of QS-regulated genes. These changes are likely to influence the intra- and inter-strain interactions. As a result, QS inhibition can introduce changes into the microbiome by redistributing the competitive advantage during the development of a complex community.

A most striking example lies in *Pseudomonas aeruginosa*, a common human pathogen that can cause infections in cystic fibrosis (CF) patients. In *P. aeruginosa*, the QSI pressure (*in vitro* and *in vivo*) can lead to the selection of QS-negative strains. In patients with CF, loss of QS signaling is associated with chronic infection and increased growth rates^18^. On the other hand, QS-regulated virulence determinants such as elastase, rhamnolipids and alginate play a key role in establishing *P. aeruginosa* colonization in the CF lung^19^,^20^. Thus, the development of QSI-based therapies should consider how the pressure of QSIs selects for QS mutants with modifications not only in their cooperative and competitive behaviors, but also in their virulence potential. However, existing simulation tools (see Supplementary Table 1) are unable to efficiently simulate dense networks of interacting bacteria populations in a complex 3D environment and incorporate both cellular and population level dynamics among bacteria in the meantime.

To investigate the major health problem of the emergence and the spread of QSI resistance, we develop a new computational framework (see Supplementary Fig. 1) to analyze the long-term dynamics of QSI-based therapies on the development and stability of biofilms and emergence of QSI-resistance. Our model uniquely accounts for mutations in different components of the QS machinery, as well as multiple properties of QS-regulated genes. The selective pressures on the QS variable cells will depend on the accessibility of QS-metabolic products to the neighboring cells. To capture this aspect, we model four types of QS outputs: (i) non-beneficial, (ii) private, (iii) quasi-public, and (iv) public goods (detailed modeling specifics are available in **Methods**). For each scenario, we consider the pathogenic properties of strains and the probability of the emergence of QSI-resistance.

Our proposed simulation framework simultaneously considers both intracellular and intercellular signaling and its effects on biofilm dynamics. We note that the intercellular network approach we propose can quantify various type of interactions and dynamics in populations of bacteria. For conditions where QSI-resistance spreads rapidly, we demonstrate that the metabolic output (i.e., different kinds of goods) of the community can substantially alter the spread of resistance. Specifically, our simulations suggest that the quasi-public goods (e.g., extracellular polymeric substances (EPS)) plays an important role in the spread of QSI resistance. Thus, we also model drugs targeting the quasi-public and public goods, and demonstrate that if resistance to such drugs arises, it will rapidly spread in a population.

Looking through such a quantitative lens at these complex phenomena can open new avenues both in fundamental knowledge and disease treatment. More specifically, from a theoretical point of view, the new network-based approach helps us model and investigate the cell-cell interaction and biofilm dynamics; this can help us estimate how manipulating signaling pathways influences the cell-cell interactions and coordination during biofilm attachment, growth, and detachment. From a practical perspective, our model provides a platform to experiment with the evolutionary outcome of different QSI therapies (alone and in combination) and prioritize testable hypotheses for strategies that will limit the emergence of QSI resistance. Moreover, the “computational microscope”^21^,^22^ we propose offers the advantages of rapid turnaround (hours instead of days), ability to test theoretical strategies, and high reproducibility of simulations. Taken together, all these contributions help us better understand and model the spread (dynamics) of QSI resistance which is paramount to the long-term success of QSIs in treating human pathogens.

## Results

### Simulation Environment and Model Calibration

**Figure 1:**
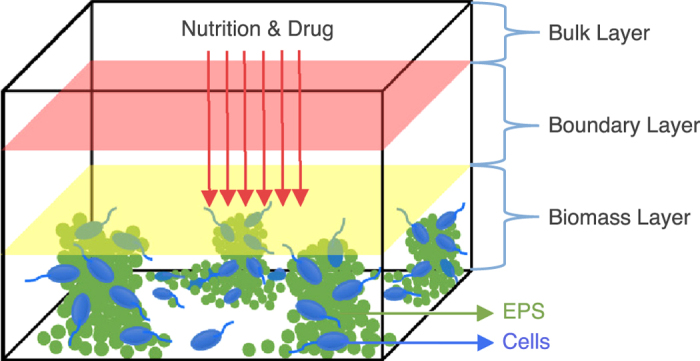
The simulation environment is modeled as bulk, boundary, and biomass layers. The chemical concentrations in the bulk layer are invariant over time. To account for the high cell/EPS density, the biomass layer has a diffusion coefficient value roughly half of the boundary layer. More precisely, since the viscosity in high cell/EPS density is lower, we assume that the diffusion coefficient is half compared to the boundary layer. Bacterial cells attach to the substrate and grow upwards with nutrition diffusing from the bulk layer toward the biomass layer. See biofilm formation in Supplementary Videos 1 and 2.

We explain the model of the cell-cell interactions in **Methods** and define a 3D microfluidic environment as shown in Fig. 1. We note that our 3D simulator accounts for the spatial volume occupied by bacteria. In other words, we can capture the dynamics of interacting particles, while taking into account that a certain spatial volume can be occupied by at most one particle at any given point in time. (The details of various simulation environment configurations are described in Methods).

Experiments were performed with a dual labeled Pseudomonas aeruginosa PAO1 strain, generously provided by Michael Givskov^23^. The strain encodes a chromosomally integrated lasB-gfp(ASV) reporter system, as well as rfp under the control of a constitutive promoter^23^. Thus, the strain produces rfp under all conditions, and gfp when QS is active. The growth condition and strain are described in Methods.

**Figure 2:**
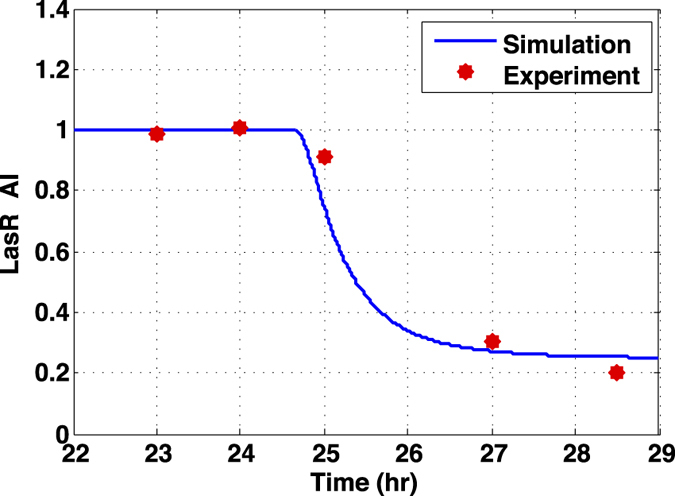
Simulation-model calibration. Confocal images where all bacteria expressed red fluorescent protein (red), and only the subset undergoing QS express green fluorescent protein (gfp) were used to measure the influence of C-30 Furanone (an AHL analog) on the ratio of signaling cells in a biofilm (red circles represent measured ratios). The QS signaling decreases over time, and disappears 4 hrs post-treatment. We calibrate our model to these experimental results.

We calibrated our QS and QSI models with experimental data. More precisely, we added Furanone at a concentration of 100 uM at time equal to 24 hr (1 day after biofilm seeded), and imaged biofilms at 25, 27 and 28.5 hr. Images were processed to obtain the percentage of total cells positive for QS signal (see **Methods**), and this data was used to calibrate the model parameters (see Fig. 2 and Supplementary Table 2).

### Selection of the Bacterial Genotypes and Regulons

**Figure 3:**
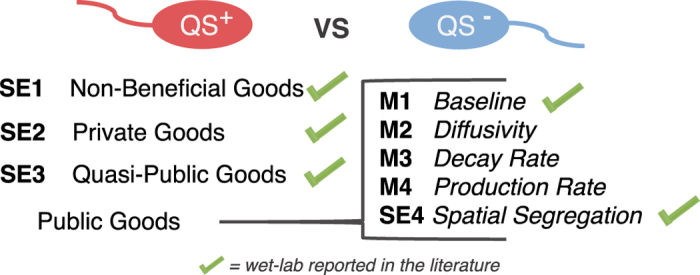
Simulation roadmap for our initial experiments on *QS*^+^/*QS*^−^ competition *without* QS inhibition. Each experimental setup (i.e., SE1-4, M1-4) is characterized by the class of goods generated by the *QS*^+^ strains and/or environmental conditions. Our simulation experiments, which corroborate previously reported wet-lab results, are indicated by green checkmarks.

We define three bacterial genotypes: (i) functional QS systems sensitivity to QSI (*QS*^+^), (ii) deletion of the QS machinery (*QS*^−^), (iii) or modified QS systems that are functional but resistant to QSIs (QSI-resistant). Additionally, to account for the production of multiple types of goods (Fig. 3), we classify the bacterial regulons into four categories: (i) non-beneficial goods, (ii) private goods, (iii) quasi-public goods, and (iv) public goods. Details are described in **Methods**.

### Simulations *without* QSI under Different Conditions

The first scenario (Supplementary SE1) we corroborate with simulations is the non-beneficial goods, where *QS*^+^ cells suffer a metabolic cost and display a growth disadvantage over *QS*^−^ cells (Supplementary Fig. 2). This has been observed with pathogenic *E. coli* strains expressing virulence factors, such as *Shiga* toxins, that do not appear to confer a fitness advantage in human infection^24^.

Next, we test private goods (Supplementary SE2), which are only available to producing cells. We confirm the relative fitness of *QS*^+^ cells over *QS*^−^ cells (Supplementary Fig. 3). It has been reported that, in the presence of the carbon source adenosine, *P. aeruginosa* is only able to gain access to nutritional benefits if the adenosine is first metabolized by the degradative enzyme nucleoside hydrolase (*Nuh*) that is positively regulated by *LasR*. *Nuh* is considered a private good as it is only available in the periplasm of the producing cell, thus conferring a metabolic gain solely to the individual cells^25^.

Finally, our simulations correctly capture a case of quasi-public goods (Supplementary SE3). A prime example of quasi-public goods is the EPS secreted during biofilm formation. The QS-mediated production and secretion of EPS provides structural support to the extracellular matrix of a developing biofilm that, in turn, provides insulation from external threats, as well as increased access to nutrients^26^,^27^,^28^. Although metabolically costly to its *QS*^+^ producers, EPS confers a large competitive advantage due to its hardly exploitable nature beyond the immediate vicinity of the producers. As shown in Supplementary Fig. 4, the biofilm growth trajectories show that the EPS cells (yellow spherical globules) surround *QS*^+^ and provide biofilm structure support. Additionally, *QS*^+^ cells gain a competitive advantage by jettisoning themselves and their lineages into higher nutrient rich regions while suffocating *QS*^−^ cells below. Our simulation results capture the relative fitness of *QS*^+^ cells over *QS*^−^ cells if the quasi-public goods are hardly exploitable for non-producer cells (*QS*^−^ cells).

In the following subsection, we provide three scenarios (M2-M4) where we modify diffusion, decay, and production rates of public goods.

### The Effect of Public Good Properties on Biofilm and Network Parameters

**Figure 4:**
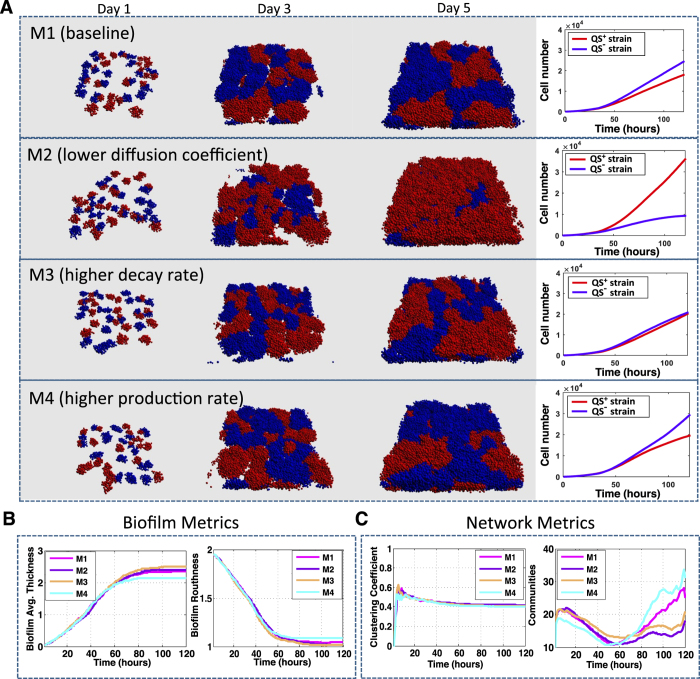
The effect of public good properties on biofilm and network parameters. We have simulated a slower molecular diffusion rate (M2), a faster public goods decay rate (M3), and higher production rate for public goods (M4) and then compared our results against the baseline (M1). Panel (A) shows projections of biofilm growth and the relative ratio of *QS*^+^ and *QS*^−^ strains over time. Panel (B) shows the physical metrics that measure the thickness and the roughness of the total biofilm with both strains combined. Panel (C) shows the network metrics that quantify the changes of the biofilm structure. Strains are color coded as red: *QS*^+^ and blue: *QS*^−^. Simulations with baseline, different diffusion rates, decay rate, and production rate are color coded as magenta, purple, orange, and cyan, respectively.

Active QS is often associated with pathogenesis, given the QS-mediated production of virulence effectors. The spread of *QS*^−^ (cheater) cells, in the presence of public goods, decreases *QS*^+^ cells and has been proposed as an anti-virulence strategy^25^. In the baseline case, the *QS*^−^ cells can take advantage of the public goods produced by *QS*^+^ cells and grow significantly faster after bacteria enter the exponential growth phase (i.e., after time t = 50 hrs), when QS controlled public goods are massively produced (Fig. 4A, M1). The biofilm metrics display the increase in thickness and decrease in roughness that occurs when QS-controlled public goods are massively produced (Fig. 4B, magenta). As the *QS*^−^ cells gain the growth advantage over *QS*^+^ cells, they split apart large communities into small communities among *QS*^+^ cells (Fig. 4C, magenta). The spread of *QS*^−^ cells has been observed both *in vitro*^29^, as well as within hosts in animal-infection models^29^. More recently, the loss of such social behavior has been identified as a consistent trend in CF lungs chronically infected by *P. aeruginosa*^30^,^31^.

We compare the baseline with the influence of decreased diffusion coefficient (Fig. 4A, M2), increased decay rate (Fig. 4A, M3), and increased production rate (Fig. 4A, M4). The two latter modifications have only mild effects on the ratio of strains and the overall biofilm structure, and lead to a decrease in the number of communities (Fig. 4B and C, orange and cyan). In contrast, decreasing diffusion rate leads to an increase in *QS*^+^ cells (Fig. 4A, M2), and fewer communities of larger size in the population (Fig. 4C, purple). This suggests that environmental changes (e.g., EPS or host immune cells) that result in a decreased diffusion of QS-public goods will increase *QS*^+^ cells. Early in colonization of CF lungs, such a change would be predicted to increase pathogenesis.

### Simulations *with* QSI Effects and QSI Resistance

**Figure 5:**
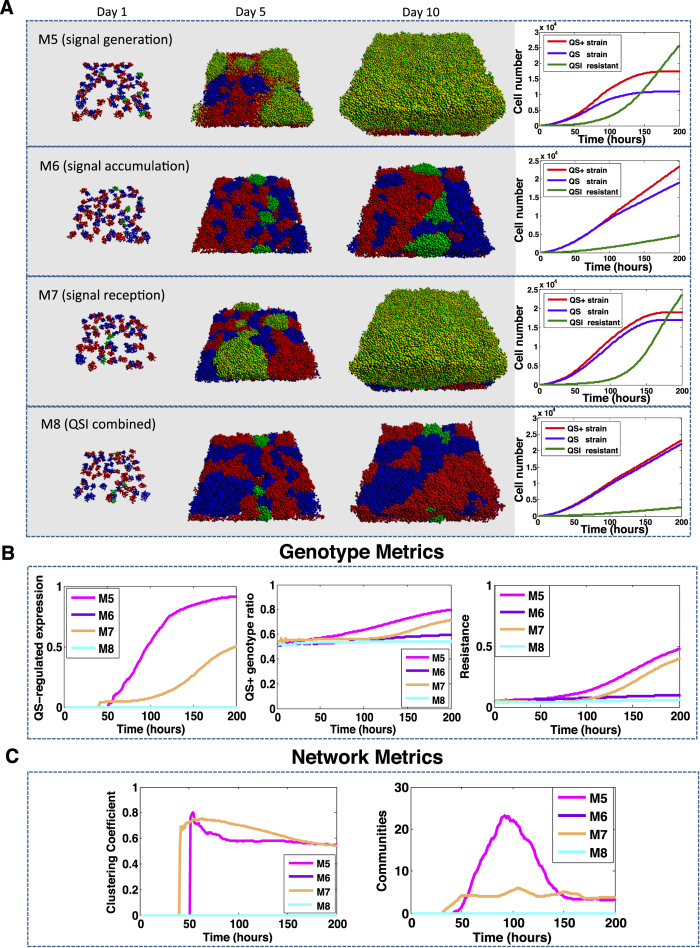
Simulations of QSI effects and spread of QSI resistances modeling both quasi-public and public goods. Four QSI targets are considered: Signal generation (M5), signal accumulation (M6), signal reception (M7), and all three combined (M8). Panel (A) shows the projections of biofilm growth up to 10 days and the dynamics of relative ratio of each strain over time. Panel (B) compares multiple QS and bacteria population measurements. Panel (C) shows the network metrics that quantify the changes of the biofilm structure. Strains are color coded as red: *QS*^+^, blue: *QS*^−^, green: QSI-resistant. Simulations with different strategies are color coded as magenta (M5), purple (M6), orange (M7), and cyan (M8).

We have developed an intercellular network model (see **Methods**) to understand the emergence and dynamics of QSI-resistance; thus, we explore three QS inhibition strategies, which target different components of the QS system^32^,^33^,^34^: (1) signal generation (Fig. 5A, M5); (2) extracellular signal accumulation (Fig. 5A, M6); (3) signal reception (Fig. 5A, M7); as well as all three treatments combined (Fig. 5A, M8). To simulate the long-term QSI treatment expected in clinical settings, the concentration of each QSI in the bulk layer (see Fig. 1) is kept constant after its introduction. Initially, 100 *μM* of QSI is added at time *t* = 0 hrs to inhibit QS and subsequent QS-mediated biofilm formation. These simulations assume QS directly regulates the production of private goods, quasi-public goods, and diffusible public goods. The simulations are performed with each type of QSI. We model QSI-resistant cells to intracellular *signal reception* and *generation*, but not to extracellular signal degrading enzymes, such as lactonase. We also run experiments to independently target non-beneficial goods, private goods, quasi-public, and public goods (Supplementary Note 2–4, Supplementary Figs 6–8).

When the model includes the production of both quasi-public and public goods, QSI spreads if the drug targets either signal generation (Fig. 5A, M5) or signal reception (Fig. 5A, M7). Importantly, this spread is *not* observed if QS leads only to production of public goods (Supplementary Fig. 8). The QSI-resistant cells begin to generate QS-regulated proteins to boost their growth around time *t* = 50 hrs (Fig. 5A). At this point in time, the communication between communities increases dramatically and stabilizes over time (Fig. 5C, magenta and orange). In both cases the number of communities stabilizes after *t* = 150 hrs, the QSI-resistant cells outcompete the other two strains, organize into high number of communities with high connectivity, and become the dominant population (Fig. 5A, M5, M7 day 10 and Fig. 5C).

While the 10-day outcome is very similar for both of these targets, the dynamics are different. More precisely, when targeting signal production, the signal molecules produced by the QSI-resistant cells diffuse out and activate *QS*^+^ cells nearby. The *QS*^+^ cells gain an advantage over the *QS*^−^ cells (Fig. 5, M5 day3). This advantage leads to formation of small clusters of *QS*^+^ cells (observed as hill-like shape in the number of communities in Fig. 5C, magenta line). In the long-term, this slow quasi-public goods production at boundaries fails to endow a fitness advantage when competing with QSI-resistant cells, and the many communities join the resistant cell communities (observed as drop in the number of communities in Fig. 5C at time *t* = 150 hrs). In contrast, when targeting signal reception, the *QS*^+^ cells do not encounter this temporary advantage over *QS*^−^ (Fig. 5A, M7). In both scenarios, when our model includes EPS, it predicts that targeting signal generation or reception may lead to the spread of QSI resistance.

When using signal degrading enzymes in M6, the QSI-resistant strains do not spread. The intracellular resistance that we model does not rescue cells from the extracellular degradation. In this scenario, we observe similar outcomes for QSI-resistant and *QS*^+^ cells. The total number of QSI-resistant cells is lower only because it reflects their lower number at time *t* = 0 hrs (see Supplementary Fig. 10 and Supplementary Note 5 for model robustness). When combining all three drug classes simulations resemble the one observed when signal accumulation is targeted. These simulations suggest targeting signal accumulation could be an effective long-term strategy for infection control.

To our knowledge, this is the first time when simulations have been used to estimate the spread of resistance for QSIs targeting signal production and reception. We observe that resistance to drugs targeting the production and reception of QS spreads rapidly in our models if they include quasi-public (EPS) and public goods in the meantime (Fig. 5, M5 and M7 respectively). In contrast, QSI resistance does not spread in our models that account only for public goods (Supplementary Fig. 8, S11 and S13, respectively). These differences highlight the important role of EPS in the spread of QSI resistance.

### Simulations of drugs targeting the production of public or quasi-public goods

**Figure 6:**
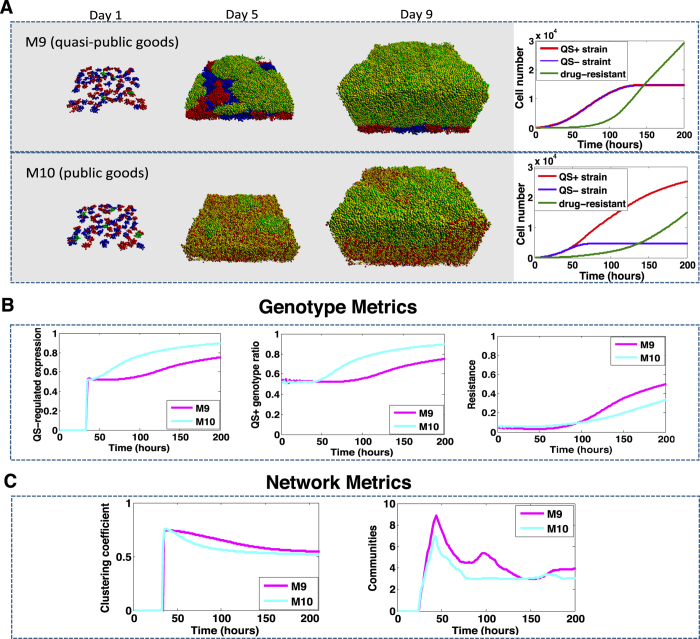
The spread of resistance to inhibitors of QS products. *QS*^+^, *QS*^−^ and drug resistant strains are treated with an inhibitor of quasi-public goods (M9) or an inhibitor of public goods (M10). Panel (A) shows the projections of biofilm growth up to 9 days and the relative ratio of each strain over time. Panel (B) compares multiple QS and bacteria population measurements. Panel (C) shows the network metrics that quantify the changes of the biofilm structure. Strains are color coded as red: *QS*^+^, blue: *QS*^−^, green: drug resistant. Simulations with different strategies are color coded as magenta (M9) and purple (M10).

Targeting quasi-public goods (i.e., EPS) has been explored as an antimicrobial strategy, given the importance of EPS in biofilms^35^. Thus we model the spread of resistance to therapies targeting enzymes involved in production of public or quasi-public goods. We test two putative inhibitors, one that targets quasi-public goods such as the biofilm matrix (Fig. 6A, M9) and another that targets public goods (Fig. 6A, M10).

Under both therapies modeled, the drug resistant strains spread rapidly throughout the biofilms (Fig. 6A). The difference between inhibitors specifically targeting quasi-public versus public goods is the fitness of *QS*^−^ strains, and the rate at which resistant strains expand in the population (Fig. 6A,B). Specifically, when quasi-public goods are inhibited both *QS*^−^ and *QS*^+^ cells exhibit similar growth rates, and the spread of resistant strains is higher. When public goods are inhibited, the number of *QS*^−^ cells is lower than that of *QS*^+^ cells. This difference is explained by the fitness costs incurred by *QS*^−^ strains in the absence of access to quasi-public goods. However, in all cases the biofilm structure exhibits similar patterns, defined by a peak in the number of communities at the time point of QS production (*t* = 50 hrs), and a stabilization of larger resistant communities after 3 days (Fig. 6C). The spread of resistance and the community of resistance strains suggest that once resistance to such inhibitors arises, it will spread rapidly throughout the population.

## Discussion

**Figure 7:**
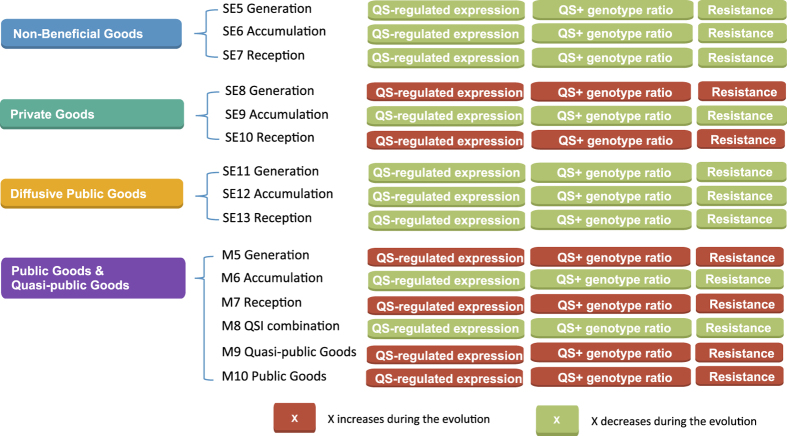
Simulations summary. The conditions and factors of each *in silico* experiment are outlined and labeled with respect to the successful decrease in QS regulated expression, *QS*^+^ genotype ratio, QSI resistance (SE5-SE11, and M5-M8), and drug resistance (M9 and M10).

In this paper, we have explored, through comprehensive computational studies, the effects of QSI on multi-strain biofilms consisting of *QS*^+^, *QS*^−^, and QSI-resistant cells, and the proliferation of resistance throughout a bacterial population. To this end, we have considered properties of molecules in the QS regulon, including non-beneficial goods, private goods, quasi-public goods, and public goods. Further, we have considered three distinct classes of QSIs targeting signal generation, signal molecule accumulation, and signal reception (Fig. 7).

One of the main contributions of this paper is the introduction of a network-theoretic framework to describe and analyze the “social” collaboration among bacteria within a population. We have applied network metrics such as the average clustering coefficient and the number of communities to understand the spatial distribution of strains with variation in QS during biofilm development. Specifically, we have used the network clustering coefficient to describe the size and distribution of clusters composed of cells with the same genotypes. By tracking the dynamic status of the network, one can observe how different drugs (see Figs 5 and 6 and Supplementary Figs 6–8), such as QSI with different QS component targets, work on the biofilms in *real-time*, thus, unveiling the underlying bacterial social interactions. We also note that in contrast to the physical biofilm metrics (e.g., biofilm average thickness and biofilm roughness), the network metrics can best discriminate the spread of QS mutants. Indeed, as shown in Fig. 4C, the number of communities show different behaviors of different biofilm organization based on properties that modify diffusible goods. While these parameters are easily outputted in our model, it is challenging to obtain similar metrics in laboratory experiments.

From a biological standpoint, our work suggests that when the QS regulon controls quasi-public goods (such as EPS), and QSI targets either signal generation or reception, once emerged QSI-resistance will rapidly spread (Fig. 5, M5 and M7). The spread is not observed if network links are completely dissolved by targeting signal accumulation in the absence of QSI (Fig. 5, M6 and M8). Additionally, the spread of QSI is not observed when only public goods are considered (Supplementary Fig. 8). If QSI or inhibitors of QS products are employed separately, both are prone to the spread of resistance (Figs 5 and 6). But, if QSI is combined with an EPS-targeting drug(s), the spread of resistance may be slowed or contained. Currently, EPS targeting can be achieved by expressing the degradation enzymes or adding compounds that act directly on the EPS (e.g., eDNA^36^). This modeling suggests that the best long-term strategy to avoid QSI resistance while combating virulence associated with QS, is to combine QSIs and EPS targeting drugs. This opens the door for future experiments, testing the spread of QSI resistance in the presence of QSI and anti-EPS drugs, where the drug-induced absence of EPS is predicted to decrease the spread of QSI resistance.

Most QSI resistance mechanisms reported so far are based on multidrug efflux pumps or cellular enzymes^37^. Resistance to signal degrading enzymes, such as the lactonase that hydrolyzes the ester bond of the homoserine lactone ring of AHLs, has not been reported so far. Thus, the lack or resistance spread observed with lactonase (Fig. 5, M6) and combinations of QSI (Fig. 5, M8) may reflect clinical scenarios. However, it is conceivable that resistance to lactonases will emerge, or is already present in the environment, and will spread to the human microbiome.

The cheater behavior of *QS*^−^ cells, and their delay on population growth has been reported, consistent with our model for QS in the context of public goods^38^. However, our simulation results contradict another scenario presented in ref. 38 where resistant strains did not spread in the presence of QSI. However, in this scenario the native QS levels were below the QS activation threshold. Our model represents a scenario where the QS signal is above the activation threshold.

Our computational approaches offer a tool to guide experimental design by generating hypotheses on drug combinations, timing, and conditions that either increase or reduce the spread of drug resistance. The simulation results suggest a combined therapy targeting quasi-public goods and QS. Additionally, with the increasing development of more efficient, accurate, and inexpensive medical technologies (cutting-edge omics technologies such as metagenomics), it is now possible to measure evolution dynamics of biofilm *in vitro*. To this end, our simulation models can be validated and verified. Overall, this work computationally explores the QSI resistance, and predicts the timing and targets for therapeutics that may decrease or inhibit the spread of QSI resistance.

## Methods

### Bacteria Strain and Growth Condition

Bacterial Strain: We utilized the dual labeled *Pseudomonas aeruginosa*, with a QS reporter. The strain, PAO1(wt)::lasB-gfp(ASV)::Plac-lasR::rfp, was generously provided by Michael Givskok^23^. Furanone C-30 was acquired from Sigma-Aldrich Co., and used at a final concentration of 100 *μ*M.

*P. aeruginosa* biofilms: Biofilms were set up on MatTek plates, at 37 °C using Columbia browth. Furanone was added at time of 24 hour after biofilm development. Biofilm images were collected on a Zeis510 Meta Conforcor3 Laser Scanning Microscope at 25, 27, and 28.5 hours post-seeding.

### Bacterial Image Processing

We detect the intensity of each bacterial image acquired from the confocal laser. Next, we compute the number of bacteria that express rfp and gfp, respectively. We then compute the relative concentration of the QS signal by using the following formula:


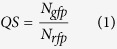


where *N*_*rfp*_ and *N*_*gfp*_ represent the number of bacteria that express rfp and gfp, respectively.

### Definition of the Bacterial Genotypes and Regulons

- **Bacterial genotypes:** Specifically, we make use of three naturally occurring genotypes in regards to QS genes^39^,^40^ (i) functional QS systems sensitivity to QSI (*QS*^+^), (ii) deletion of the QS machinery (*QS*^−^), (iii) or modified QS systems that are functional but resistant to QSIs (QSI resistant). *QS*^−^ cells that have access to the benefits of QS, but do not incur the metabolic costs of production, should be at an adaptive advantage relative to their *QS*^+^ counterparts and can be characterized as “cheaters” within the community. We assume that the strains do not differ in other regions, and consequently there are no fitness advantage incurred by variability in other genomic regions.
- **Bacterial regulons:** Signaling via QS can lead to the production of multiple types of goods (Fig. 3). We separate these into four categories: (i) non-beneficial goods that provide no fitness advantage to producers or neighboring cells; (ii) private goods that provide a fitness advantage to producers but not neighboring, (iii) quasi-public goods that provide a fitness advantage to producers and neighboring cells but remain exclusively accessible to their producers, and (iv) public goods that provide a fitness advantage to producers as well as neighboring cells. The competitive index between *QS*^+^ and *QS*^−^ depends the production costs and benefits of QS, and the availability of QS products to *QS*^−^ cells.

### Bacteria Population Model

The intracellular biochemical pathways giving rise to quorum sensing have been well studied from both molecular and systems biological perspectives^41^,^42^,^43^,^44^. Yet, the endogenous mechanisms of such signaling systems are inextricably bound to exogenous processes, cues, and constraints suggesting that, in isolation, intracellular models are unrealistic^45^,^46^. Consequently, recent efforts have been put forth to contextualize the intracellular signaling mechanisms within the scope of intercellular interactions^47^,^48^,^49^,^50^,^51^,^52^,^53^,^54^,^55^. Nevertheless, this line of work has, hitherto, been limited to interactions among small groups of cells and has neither considered characterizations of molecular communication at a higher level of abstraction, nor differentiated cell types capable of behavioral variances including cooperation, exploitation, and pathogenesis.

To this end, we present a new computational model for bacterial community dynamics capable of capturing highly emergent behaviors found at the population level. We draw from the paradigm of agent-based modeling whereby each cell is outfitted with its own set of equations governing metabolic and communication processes^56^,^57^,^58^,^59^,^60^. Our model domain consists of a 3-dimensional micro-fluidic environment governed by the laws of diffusion and volume exclusion. The model incorporates molecular signaling, nutrient limited metabolism, exoproduct formation, and virulence processes that are crucial in the development and evolution of mixed biofilm communities (see Supplementary Table 2 for model parameters).

In our model, we assume the QS regulatory network of *Pseudomonas aeruginosa* has two feedback loops (see Supplementary Fig. 9). The *LasR* − *AHL* complex up-regulates the expression of both *lasR* and *lasI* genes, generating even more signal molecules (*AHL*) and receptors (*LasR*), thereby forming a positive feedback loop. To model the QS system, we use a set of ordinary differential equations proposed in refs 47 and 61, and then extend the ODE system to incorporate the effects of QSI. Particularly, we focus on three components of the QS system that are potential targets for QSI therapy: (1) *LasI* signal synthase (i.e., signal generation) (2) Extracellular *AHL* molecules (i.e., signal accumulation) (3) *LasR* antagonist (i.e., signal reception)^32^,^33^.

Furthermore, we simulate bacterial growth kinetics based on the classic Monod model^62^ by considering multiple nutrient sources. The production of all inter or intracellular proteins are all constrained by nutrition availability in the ambient microenvironment. Nutrients and drug molecules diffuse across the space following Fick’s low while using different diffusion coefficient for different layers (see Fig. 1). Physical collisions are handled using CUDA platform which is capable of calculating the overlapping area of thousands of particle pairs (i.e., cells and EPS) and shoving them simultaneously.

### Quorum Sensing Model

The regulatory network of the *LasR*/*R* quorum sensing system has two feedback loops. Based on the ODE-models proposed in refs 47 and 61, we have the following equations for the *luxIR* QS system:


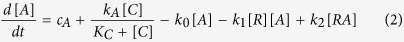



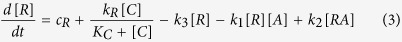



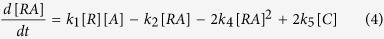



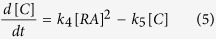


where [*X*] denotes the concentration of a particular molecular species *X*. In our formulation, *A* stands for *AHL*, *R* is *LasR* homologs, *RA* is the *LasR* − *AHL* complex and *C* is the dimerized complex. *c*_*A*_ and *c*_*R*_ account for the basal level transcription of *A* and *R*, respectively. The values and references of the parameters are adapted from a general *LasIR* system^47^.

To give some intuition, the first term *c*_*A*_ of Eq.(2) describes the basal level transcription, the second term 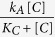 captures the positive feedback loop regulated by the dimerized complex *C* and the third and forth terms describe the *AHL* concentration changes caused by the binding and unbinding reactions of *AHL* and *LasR* receptor, respectively. The last term describes the degradation of *AHL*. Eqs (4) and (5) describe the binding reaction of *AHL* and *LasR* receptor, as well as the dimerization process of the binding product [*RA*], respectively.

### Quorum Sensing Inhibition Model

Recently, a large number of chemical compounds have been screened and many of them show QS inhibition effects targeting various components of the QS system. In this paper, we focus on three components of the QS system that are potential targets for QSI therapy: (1) *LasI* Signal Synthase (i.e., signal generation) (2) Extracellular *AHL* molecules (i.e., signal generation) (3) *LasR* antagonist (i.e., signal reception)^32^,^33^.

(1) Inhibition of *LasI* signal synthase. AHL synthase catalyses the formation of an amide bond between the homoserine lactone ring from S-adenosylmethionine (SAM) and the acyl chain from the acyl acyl-carrier-protein (acyl-ACP). After catalysed, the product of AHL and by-products of holo-ACP and 59-methylthioadenosine (MTA) are generated and released^63^. Many chemical compounds have been shown to be effective reducing *LasI* activity^14^. Recently, a new compound trans-cinnamaldehyde, which has no chemical structure similarity to AHLs or AHL analogues, is shown to inhibit AHL synthases by molecular docking studies^34^. To have a general form in our model, we modify eq. (2) to add the *lasI* inhibition; also, a new equation for the drug degradation is also added:


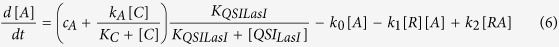



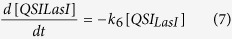


where [QSILasI] stands for the concentration of QSI molecules that inhibits *luxI* expression, *k*_*6*_ is its degradation rate.

(2) Extracellular *AHL*: *AHL* molecules can diffuse in and out of the bacterial cell freely. Therefore, once they appear in the extracellular environment, they are potential targets for destruction or inactivation. The *AHL*-degrading enzyme, *AHL*-lactonase (such as *AiiA* or *AiiM*), has been reported to deactivate the bacterial virulence through hydrolysis of the lactone ring of *AHL*^64^. Accordingly, eq. (6) needs to be modified to describe the *AHL*-degradation by *AHL*-degrading enzyme; similarly, we also need to add a new equation describing the drug degradation:


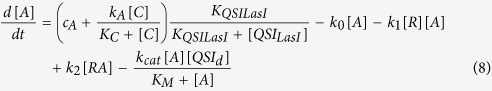



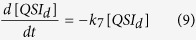


where [*QSI*_*d*_] stands for the concentration of QSI which degrades *AHL*. In these equations, *k*_*cat*_ represents the maximum rate achieved by the system, at maximum (saturating) substrate concentrations. The Michaelis constant *K*_*M*_ is the substrate concentration at which the reaction rate is half of *k*_*cat*_, and *k*_7_ is the drug degradation rate.

(3) *AHL* signal reception: The most promising mechanism for inhibiting *LasR* activation is achieved through the use of *AHL* analogues that act as antagonists for the native *AHL* (i.e., *3O-C12-HSL* for *LasIR* system). These molecules are likely to be similar in structure to the natural *AHL* and compete for *LasR*-receptors binding (some can bind covalently). Accordingly, eq. (3) needs to be modified to:


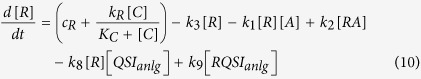


where [*QSI*_*anlg*_] stands for QSI which inhibits the *LasR* activation, [*RQSI*_*anlg*_] is the binding product of *LasR* and [*QSI*_*anlg*_]. Also, we need to add two more equations to describe the dynamics of [*QSI*_*anlg*_]:


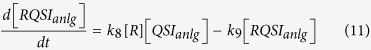



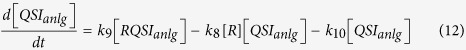


where *k*_10_ is the degradation rate of *QSI*_*anlg*_. We assume that the *AHL* analogues have the twice the affinity as the native *AHL* to the *LasR* receptor, therefore, the binding reaction rates satisfy *k*_8_ = 2*k*_1_.

### Cell Growth

Monod introduced the concept of single nutrient controlled kinetics to describe microbial growth^62^. The kinetic relates the specific growth rate (*μ*_*X*_) of a bacterium cell mass (*X*) to the substrate concentration (*S*). The kinetic parameters, maximum specific growth rate (*k*_*X*_) and substrate affinity (*K*_*S*_), are assumed to be constant and dependent on strain, medium, and growth conditions (e.g. temperature, pH). Also, we consider a second nutrient source, and add a new term *Q*. On the other hand, when cells are metabolically active, but not growing or dividing, they may still take up substrate. To address this, a maintenance rate (*m*) is generally used to describe the reduction, and Monod’s model can be improved as follows:


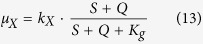



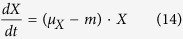


### EPS Production

We model the production of EPS as a function of the intracellular dimerized *LasR* − *AHL* complex *C* and incurring a cost on the carbon substrate (*S*) in Eq. (20):


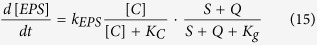


where *k*_*EPS*_ is the maximum EPS production rate, and 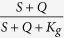 represents nutrition limitation. Using this model, we expect a maximum rate of EPS production once cells reach a quorum through communication (i.e. the level of *AHL* in the extracellular environment passes a given threshold).

### Digestive Enzyme (DGE) Production

We present a simplified and generic model of a diffusible public good motivated the production and utilization of the iron-chelating siderophore, pyoverdine. The production of *DGE* is also regulated by the intracellular dimerized *LasR* − *AHL* complex (*C*) a maximum production rate (*k*_*DGE*_),


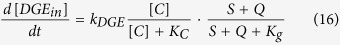


and the amount of complex *Q* (e.g., chelated iron) available in the extracellular environment is


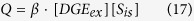


where *β* is the chelation rate, *DGE*_*in*_ and *DGE*_*ex*_ are the intracellular and extracellular *DGE* concentration, respectively.

### Metabolic Burden and Nutrition Consumption

Also, we need to take into account the cost of generating QS-related molecules, *EPS*, and digestive enzymes. Specially, to balance the limiting nutrition, we introduce utilization coefficients *U*_*X*_, *U*_*DGE*_, *U*_*EPS*_, *U*_*A*_, *U*_*R*_ to model the metabolic burden and consumption of substrate nutrition to produce general cell mass, digestive enzymes, EPS, AHL molecules, and QS receptors, respectively:


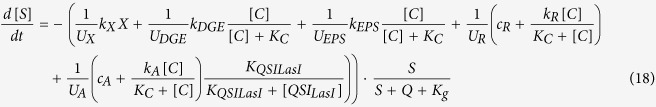



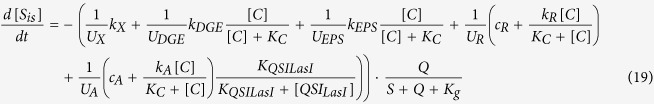


Therefore, higher cell densities can lead to a decreased growth rate as well as production rate of various cellular products in a nutrition-limited environment.

### Mass Volume, Radius, and Physical Interactions

Similar to^60^, in our simulation platform, a bacterial cell structure is compartmentalized with inner “biomass” core consisting of all intracellular material, and an outer layer consists of the capsular EPS. For each compartment, a density *ρ*_*i*_, the mass *m*_*j*_, and volume *V*_*j*_ is updated at each simulation step according to the following equation:


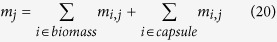



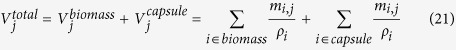


Then, the radii of the inner biomass and entire cell are calculated using the volumes *V*_*j*_ and *V*_*jtotal*_ respectively. When the volume of a cell grow to be twice of the regular size (division threshold), it divides into two daughter cells. Similarly, when the capsular mass is above a “separate threshold”, the capsular mass fall off and form a new particle, such as the EPS.

Besides chemotaxis, cell growth, division, and shrinking are all the sources of movements. We use a physical collision kernel powered by CUDA technology to resolve any pair-wise agent overlap.

### Cell-Cell Interaction Network and QS Signaling Molecules

Our network model considers the intracellular molecular information of the QS system, as well as the extracellular physical diffusion limit.

**Figure 8:**
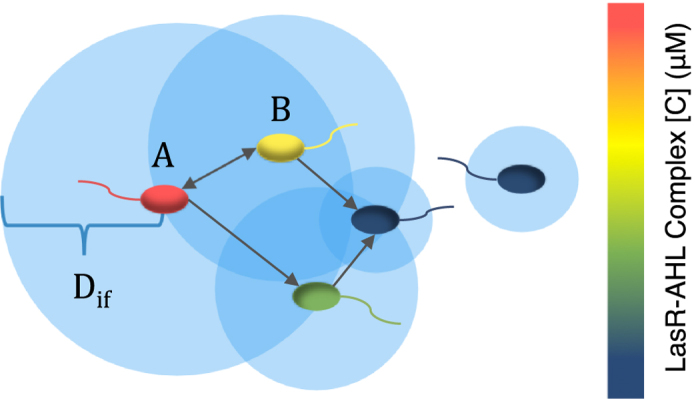
Bacteria QS-based collaboration network. A directed link is formed from cell A to cell B if cell B lies within the influence range, *D*_*if*_, of cell A and both cells have activated QS systems. The influence range, *D*_*if*_, of a cell depends largely on the intracellular concentration of the QS activity regulator complex, *C*. This is demonstrated by the bacterial color scale which represents intracellular [*C*]. The arrow from A to B indicates that A can influence B; the blue circles represent the radius of QS influence.

To replicate cell-cell interactions, in our model, a directed link from bacterium A to bacterium B is established under two conditions (see Fig. 8):

(I) Bacterium B must be within a diffusion-limited signal influence range *D*_*if*_ of bacterium A.

(II) Bacteria A and B must be QS active at the same time.

The first condition accounts for the spatially constrained nature of molecular diffusion. The signaling molecules produced and secreted by bacteria have a strong impact only within a range, *D*_*if*_, depending on production rate, diffusivity, and decay rate. The second condition ensures that the QS system of both bacteria is active; this is realized by comparing against a concentration threshold of the intracellular QS regulator. To account for different levels of signal production of spreader bacteria, *D*_*if*_ is defined as:


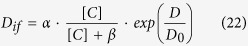


where *α* is a scaling factor for the overall influential distance, *β* is the saturation factor for the intracellular QS activity regulator complex *C*, and 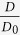 is the ratio of the effective diffusion (*D*) coefficient in the complex extracellular environment normalized to the free diffusion case (*D*_0_). It is important to emphasize that the influence range, *D*_*if*_, differs across cells as a function of intracellular QS activity regulator complex, *C*. Fig. 8 illustrates an instance of a collaboration network formed across a small number of cells.

### Physical Metrics to Quantity Biofilm Structure

- **Biofilm average thickness:** The average biofilm thickness is measured based on the distance between the substrate and the outermost cell over the space.
- **Biofilm roughness:** Biofilm surface roughness is defined by the following equation:

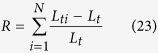


where *L*_*f*_ is the average thickness, *L*_*fi*_ is the *i*th value of the thickness matrix in the *i*th grid.

### Metrics to Quantify Interaction Dynamics and Parameters

Once the cell-cell interactions are mathematically characterized, we can derive two network metrics to describe the interaction dynamics:

- **Clustering coefficient** measures the degree to which network nodes are clustered together. In this paper, we consider a global clustering coefficient which is based on triadic network nodes^65^. Since our network links are constrained by the influential distance, a high clustering coefficient means that the network nodes are not only highly active but also in close proximity to one another, without being separated by non-active cells or other particles in the extracellular space.
- **Communities** are defined as groups of nodes with high clustering coefficient (i.e., densely interconnected) that are only sparsely connected with the rest of the network. Therefore, a cell group may have only a few links across another group, but if the two cell groups have a large impact on one another (e.g, gene expression synchronization.), then they are considered to belong to the same community.

We also define three quantitative parameters:

- **QS-regulated expression:** As indicated in our QS activity based network definition, the percentage of networked nodes (i.e. QS-activated cells) over the total number of cells (i.e., the sum of *QS*^+^ cells, *QS*^−^ cells, and *QSI*-resistant cells) can be used as a measure of the overall activity of QS-regulated product expression in a biofilm. Given that QS systems commonly up-regulate virulence factors, its expression is generally considered to be positively correlated with the percentage of networked nodes.
- *QS*^+^ **genotype ratio:** The ratio of the sum of the number of *QS*^+^ cells and *QSI*-resistant cells over the total number of cells. This measure is critical because it indicates how strong the QS activity in the bacteria populations would be once the QSI treatment is reduced or stopped, regardless of how much it is inhibited when QSI is used.
- **Resistance:** The ratio of the number of *QSI*-resistant cells over the total number of cells (i.e., the sum of *QS*^+^ cells, *QS*^−^ cells, and *QSI*-resistant cells). Note that QSI resistance is defined as the continuance of QS activity following QSI treatment.

### Simulation Environment Configuration

All the scenarios we model bacterial growth in a 3D microfluidic environment (250 *μm* × 205 *μm* × 400 *μm*) is initialized and inoculated with 25 wild-type *QS*^+^ and 25 *QS*^−^ mutant cells, all of which are non-overlapping and randomly attached to the substrate, see Fig. 1. A constant nutrition concentration, *S* = 1 *mM*, available to both types of cells is maintained in the bulk layer and free to diffuse to the biomass layer. The duration of the growth period is 5 days.

## Additional Information

**How to cite this article**: Wei, G. *et al*. *In Silico* Evaluation of the Impacts of Quorum Sensing Inhibition (QSI) on Strain Competition and Development of QSI Resistance. *Sci. Rep*. **6**, 35136; doi: 10.1038/srep35136 (2016).

## Supplementary Material

Supplementary Information

Supplementary Video S1

Supplementary Video S2
