# Supplementary material for: In Silico Evaluation of the Impacts of Quorum Sensing Inhibition (QSI) on Strain Competition and Development of QSI Resistance
Source: Sci Rep. 2016 Oct 13;6:35136. doi: 10.1038/srep35136 (PMC5062075; doi:10.1038/srep35136)
Supplement: Supplementary Information [file srep35136-s1.pdf]

In Silico Evaluation of the Impacts of Quorum Sensing Inhibition (QSI) on Strain Competition and Development of QSI resistance Supplementary Information

Guopeng Wei[1], Chieh Lo[1], Connor Walsh[1], N. Luisa Hiller[2], and Radu Marculescu[1,*]

[1]Electrical and Computer Engineering, Carnegie Mellon University, Pittsburgh, PA, USA
[2]Biological Sciences, Carnegie Mellon University, Pittsburgh, PA, USA
[*]radum@cmu.edu

Supplementary Figures

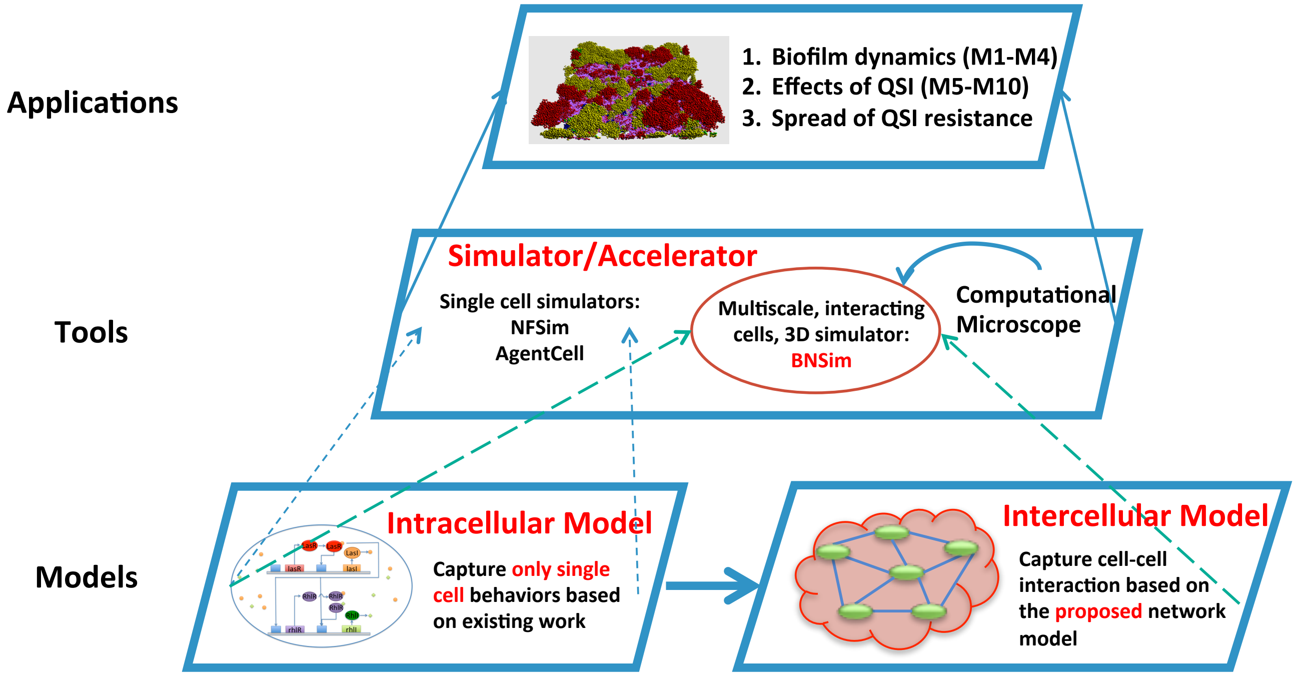

Figure 1. Fig. 1R: Our simulation framework consists of three layers. The modeling layer contains the intracellular and intercellular model; this layer provides the basis to build up the tool and application layers. One of our main contributions is to develop a cell-cell interaction model that characterizes the biofilm dynamics which are discussed in the main manuscript (the **Cell-Cell Interaction Network** under **Methods** section). Next, we run these models to capture the biofilm dynamics at ideal growth conditions (M1-M4 in the main manuscript) Finally, we model the spread of QSI resistance under different classes of inhibitors (M5-M10 in the main manuscript).

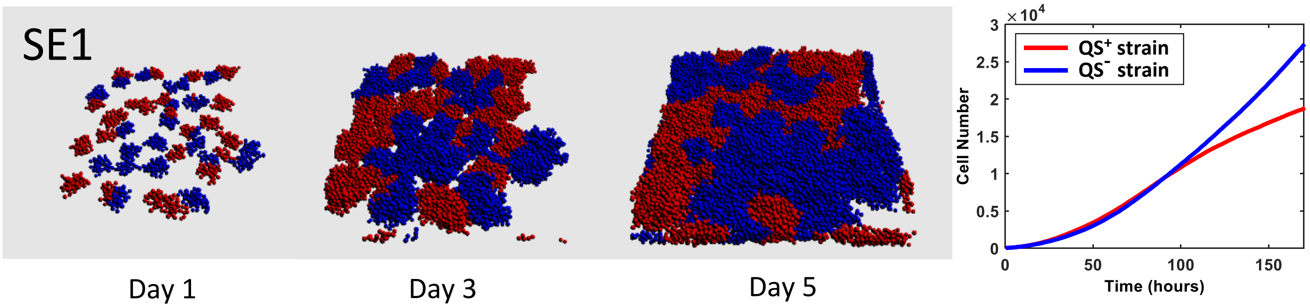

Figure 2. Bacterial growth of QS^{+} cooperators and QS^{-} cheaters where cooperators produce non-beneficial goods, and suffer strict metabolic losses. In the simulations, spherical particles are used to represent cells; QS^{+} cells are represented as red particles, QS^{-} cells are blue.

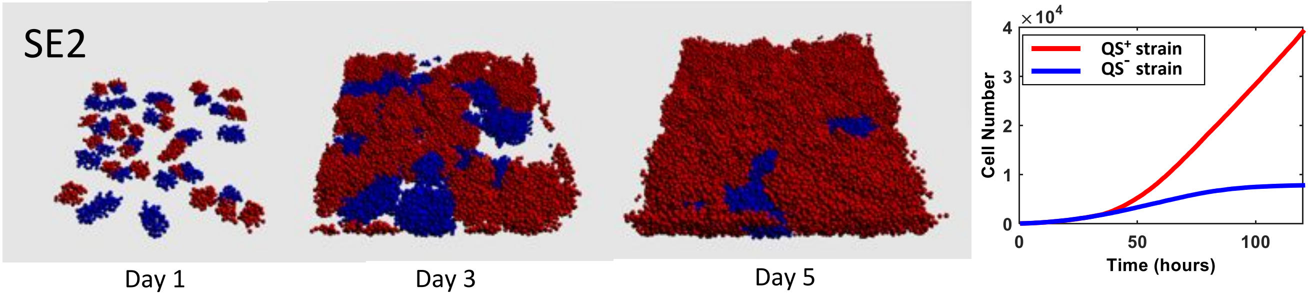

Figure 3. Bacterial growth on a secondary limiting nutrient, accessible only when metabolized by QS-mediated private enzymes within a cell. QS^{+} cells grow exponentially while the growth of QS^{-} cells is impaired. QS^{+} cells are represented as red particles, QS^{-} cells are blue.

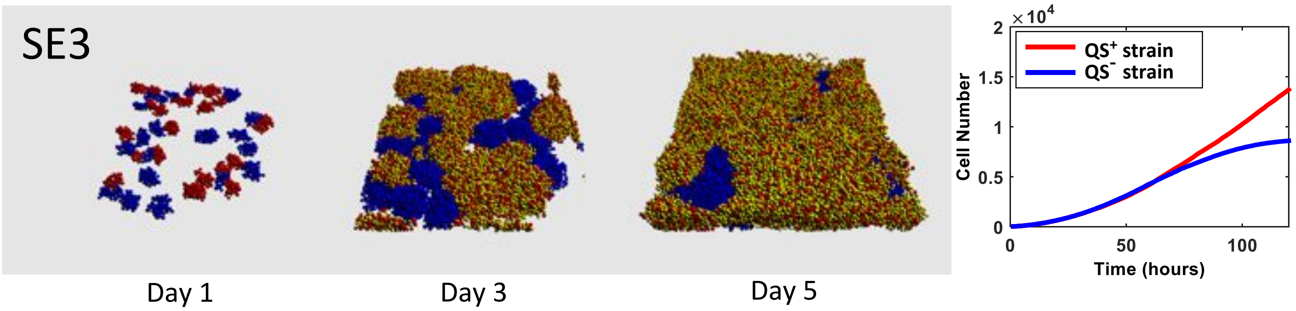

Figure 4. Bacterial growth with QS-mediated quasi-public goods secretion. QS^{+} cells gain a competitive advantage by jettisoning themselves and their lineages into higher nutrient rich regions while suffocating QS^{-} cells below. In these simulations, yellow spherical globules represent quasi-public goods which provides structural support for the biofilm.

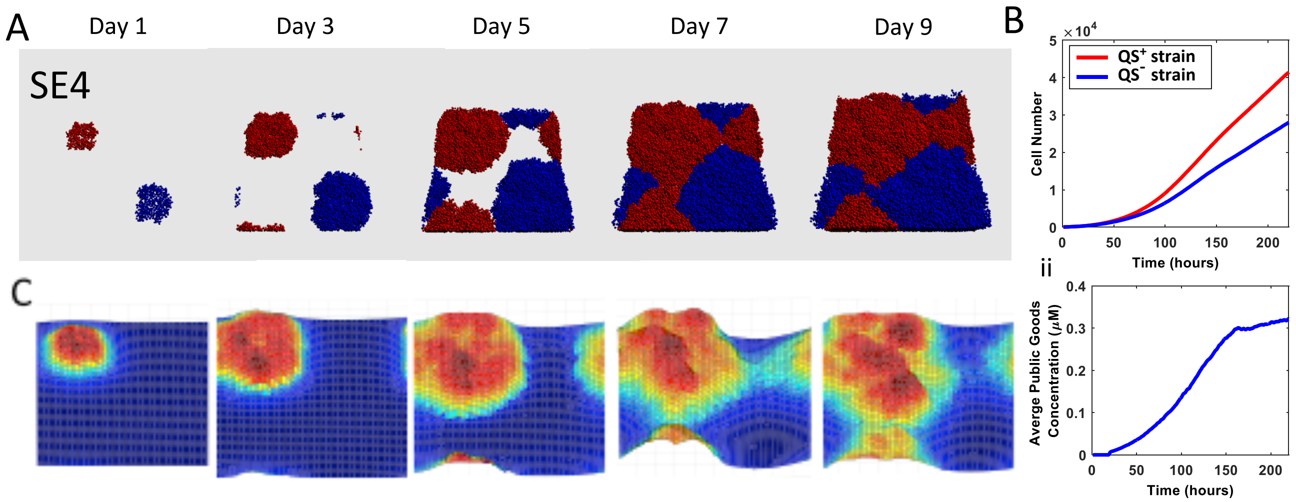

Figure 5. Bacterial growth with QS-mediated production of diffusible public goods and colonization pattern where there is physical segregation of two genotypes. The three-dimensional heat maps depict the extracellular concentration of diffusible public good and demonstrates that, while the exoproduct is freely available to cheaters, the diffusivity and spatial configuration of cooperators constrains their growth. This suggests that genotypic locality and segregation are critical in determining the exploitability of a resource.

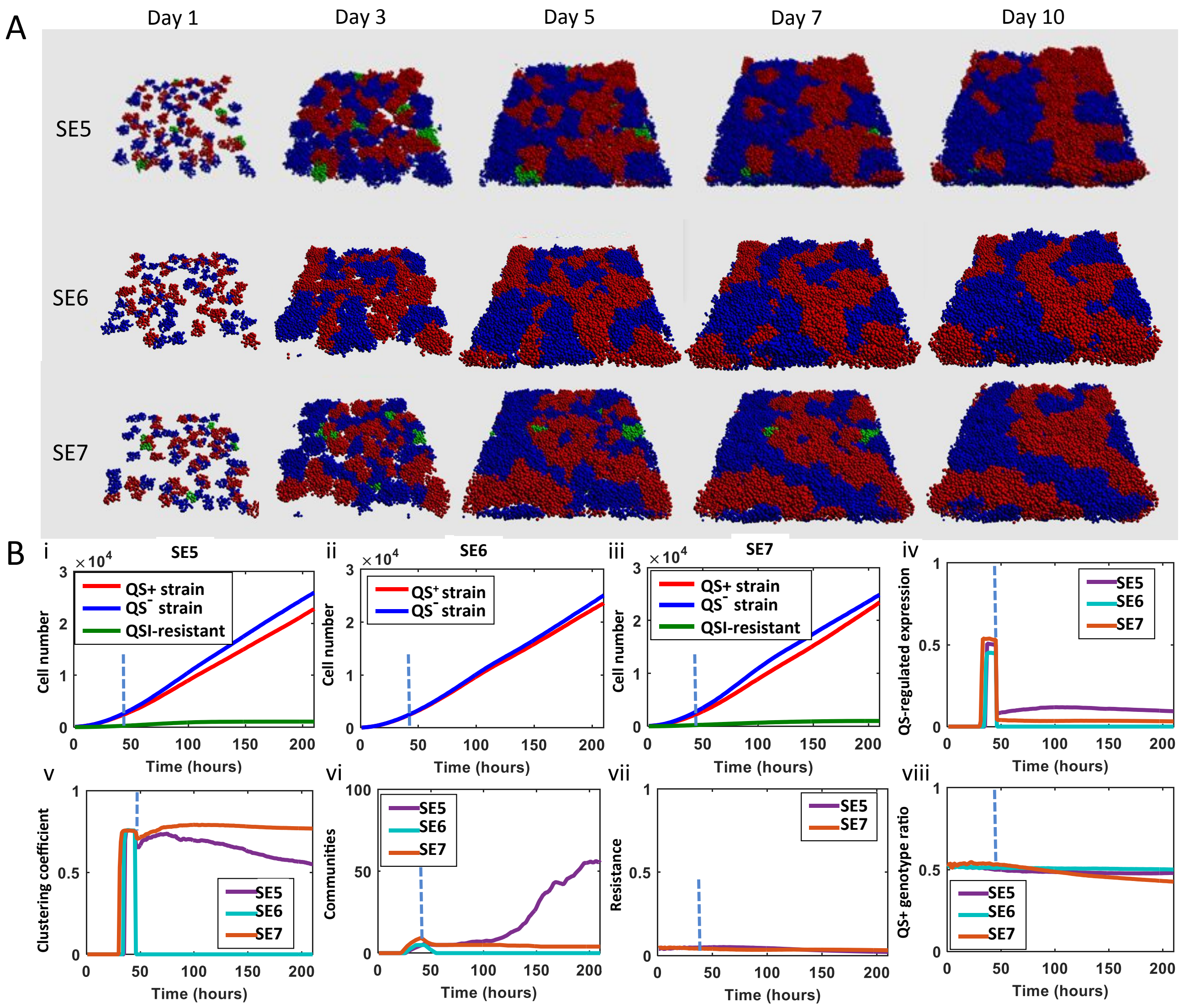

Figure 6. Dynamics of strain growth with biofilms composed of QS^{+}, QS^{-}, and QSI-resistant strains, when QS regulates the production of non-beneficial goods. Strains are represented by different colors (red:QS^{+}; blue: QS^{-}; green: QSI-resistant). Figure set A: Projections of biofilm growth up to 10 days. Figure set Bi-Biii: Graphs displaying the relative ratio of each strain, before and after QSI addition under various types of QSI (i, ii, iii correspond to scenarios SE5, SE6, and SE7, respectively). Figure set Biv-viii: Graphs comparing multiple QS and bacteria population measurements. Dotted lines corresponds to addition of QSI. Note that resistance to QSIs targeting signal accumulation is not considered, SE6 does not have QSI-resistant strain in the mix.

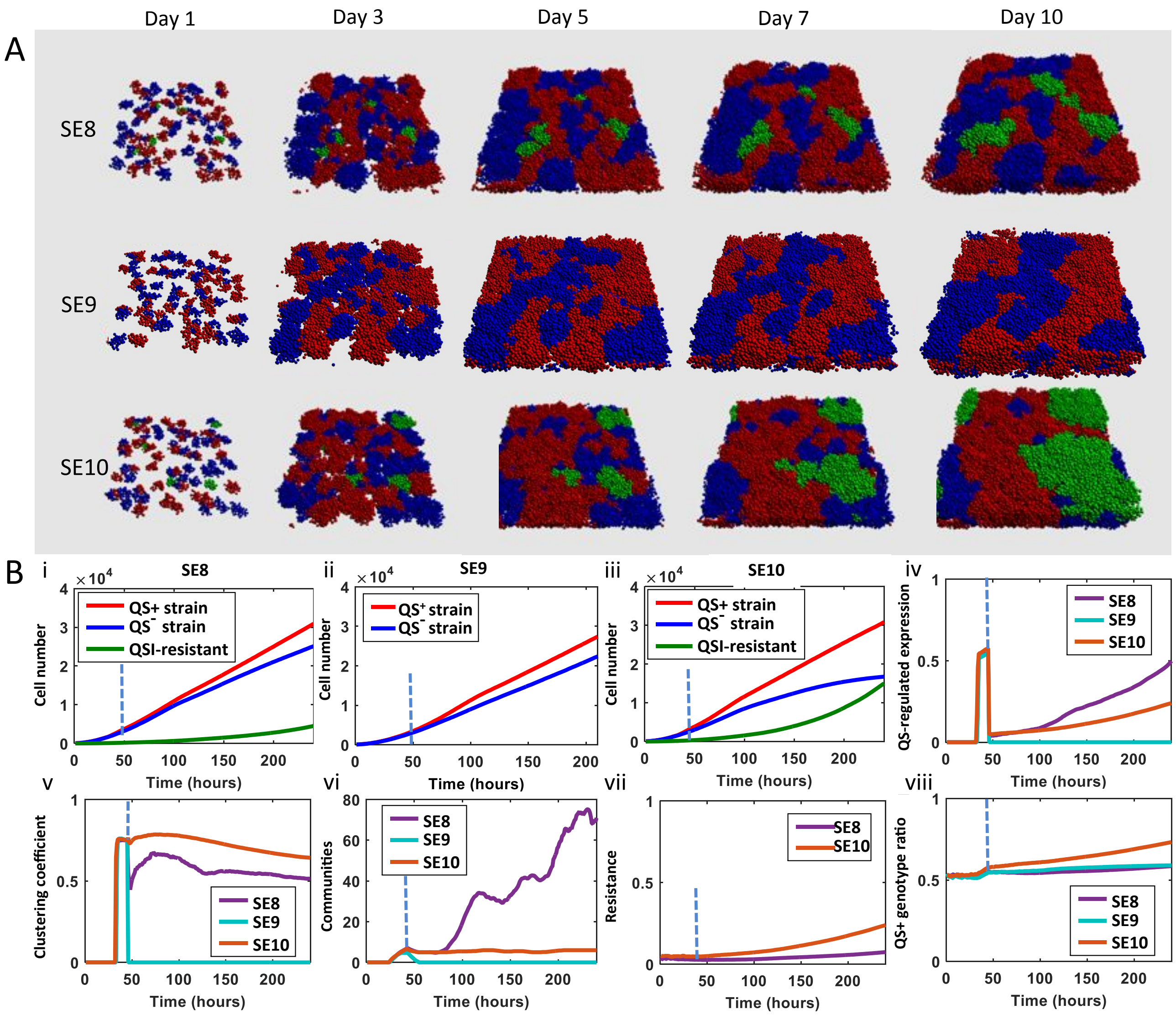

Figure 7. Dynamics of strain growth with biofilms composed of QS^{+}, QS^{-}, and QSI-resistant strains, when QS regulates the production of private goods (colored red, blue, and green respectively). Figure set A: Projections of biofilm growth up to 10 days. Figure set Bi-Biii: Graphs displaying the relative ratio of each strain, before and after QSI addition under various types of QSI (i, ii, iii correspond to scenarios SE8, SE9, and SE10, respectively). Figure set Biv-viii: Graphs comparing the consequences of different modes of QSI on multiple QS and bacteria population measurements. Note that resistance to QSIs targeting signal accumulation is not considered, SE19 does not have QSI-resistant strain in the mix.

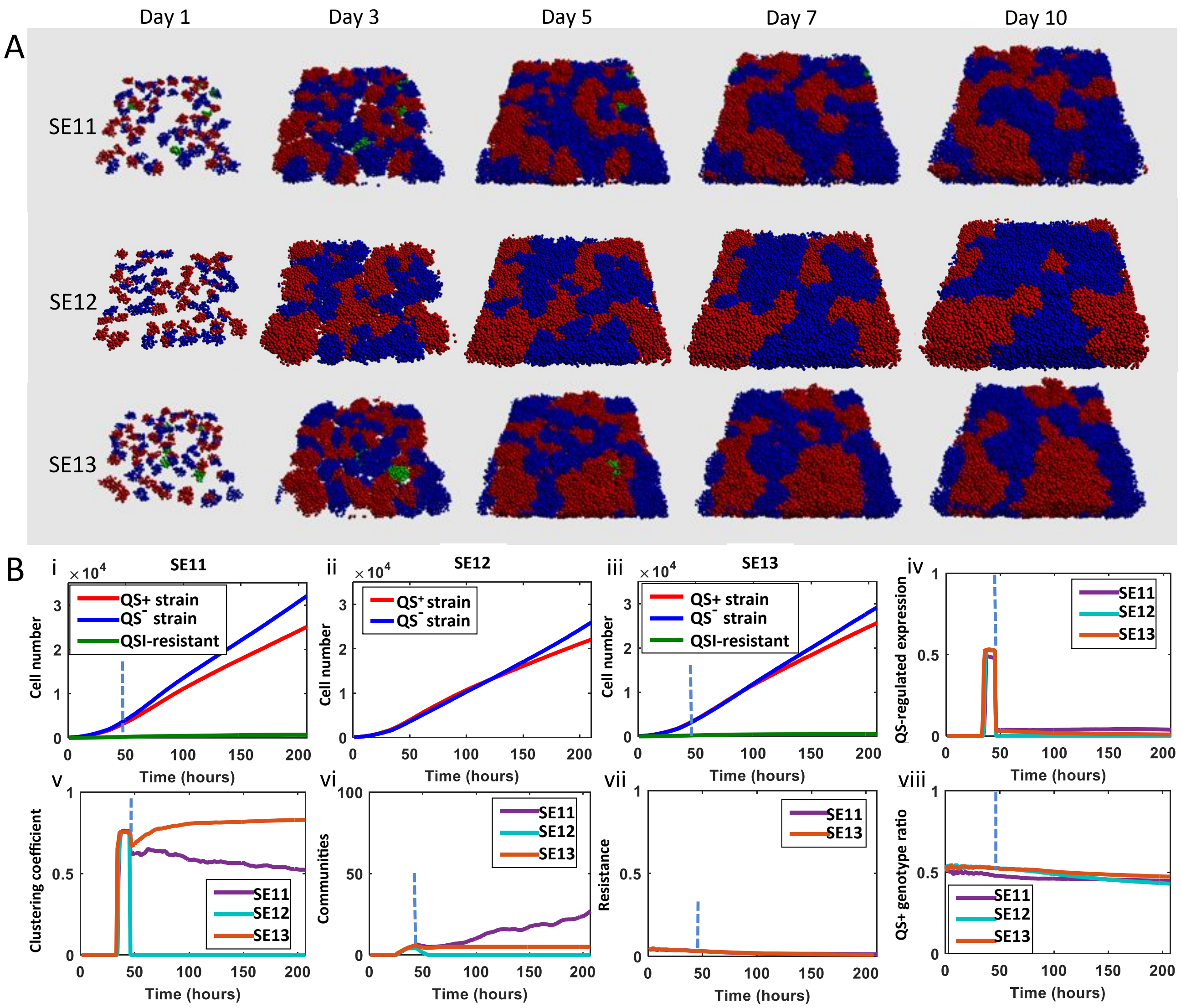

Figure 8. Dynamics of strain growth with biofilms composed of QS^{+}, QS^{-}, and QSI-resistant strains, when QS regulates the production of diffusive public goods (red, blue, and green, respectively). Figure set A: Projections of biofilm growth up to 10 days. Figure set Bi-Biii: Graphs displaying the relative ratio of each strain, before and after QSI addition under various types of QSI (i, ii, iii correspond to scenarios SE11, SE12, and SE13, respectively). Figure set Biv-viii: Graphs comparing multiple QS and bacteria population measurements. Note that resistance to QSIs targeting signal accumulation is not considered, SE12 does not have QSI-resistant strain in the mix.

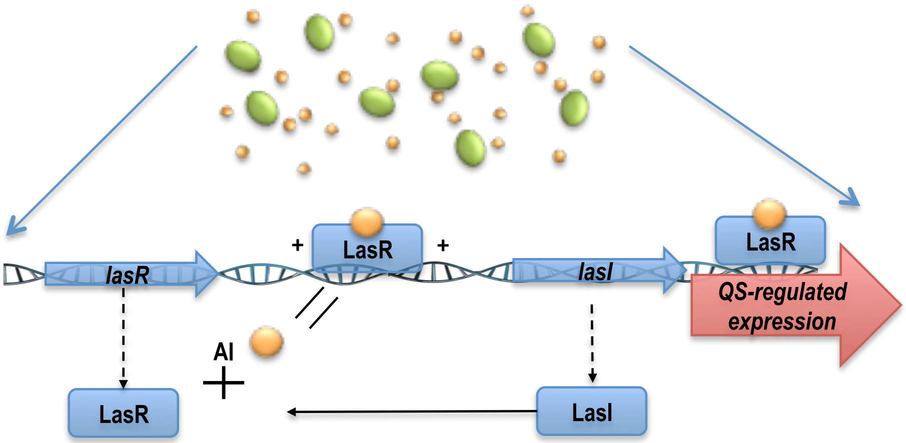

Figure 9. *Pseudomonas aeruginosa* uses $LasI/R$ quorum-sensing networks. Specifically, $LasI$ is an AHL synthase, $LasR$ is the receptor which can bind the AHL molecules, and the $LasR-AHL$ complex regulates a set of genes (red), including EPS and various virulence factors.

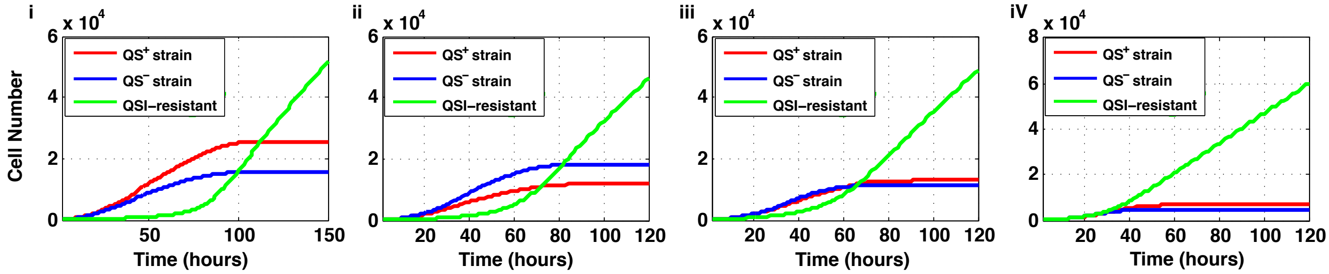

Figure 10. The evolution dynamics of different initial composition ratios of three genotypes. More precisely, the ratio of QS^{+}, QS^{-} and QSI-resistance cells is as follows: (i) 1:1:0.1, (ii) 1:1:0.3, (iii) 1:1:0.5, and (iv) 1:1:1.

Supplementary Note 1: Preliminary Success of QSIs

Previous work on using QS inhibitors can be characterized to target at least one of these three categories: (1) signal generation;[1] (2) extracellular signal accumulation;[2] (3) signal reception.[3]

For targeting on the signal generation, extensive research has evaluated the mechanisms involved in AHL production. LasI synthase proteins utilize components of the amino acid and fatty acid biosynthesis pathways to produce AHLs. Recent in vitro studies have also shown that alterations in the biosynthesis of the LasI synthase proteins can decrease the production of active AHL molecules.[1] This research data demonstrate that LasI is very sensitive to environmental conditions and that controlling the LasI substrates may have a significant effect on functional AHL production. Therefore, targeting the expression of LasI substrates can prevent the production of AHL, and thus QS activation.

For targeting on the signal accumulation, another extensively studied QSI strategy is the degradation and modification of the QS signaling molecules. Most enzymes identified thus far target AHL molecules. Lactonases degrade AHLs as they leave the bacteria, thus inhibiting their activation of LasR and host cells. Indeed, when these lactonases were expressed in *P. aeruginosa*, once can observe a significant decrease in AHL production and virulence factors expression.[2]

For targeting on the signal generation, a recent study used a reporter assay to identify a group of compounds to inhibit the activation of LasR and elastase production.[3] The antagonists are similar in structure to natural AHLs produced by *P. aeruginosa* and would compete for binding to LasR proteins; thus, inhibit the activation of LasR.

Supplementary Note 2: Simulations *with* QSI Effects and QSI Resistance, where QS Leads to Production of Non-beneficial Goods

We consider QS^+ and QSI-resistant cells produce non-beneficial goods in response to QS activation. Also, we consider three QSI strategies, namely, a) targeting signal generation (SE5), b) targeting signal accumulation, and no QSI-resistance mechanism (SE6), c) targeting signal reception (SE7).

When QS regulates the production of non-beneficial goods, the QS-regulated expression, QS^+ genotype ratio, and QSI-resistant cell frequencies do not increase; in fact, the QSI-resistant can not dominate the population in all there scenarios due to the production of non-beneficial goods has a metabolic costs on QS^+ and QSI-resistant cells, and this is not balanced by any fitness benefit. Overall, the use of QSI in the non-beneficial goods cases can effectively inhibit the QS-regulated expressions without inducing any bad outcome. That is to say, if the virulence factors are regulated by QS, and they confer no growth benefits to the producers, then QSI therapy should work very well.

Supplementary Note 3: Simulations *with* QSI Effects and QSI Resistance, where QS Leads to Production of Private Goods

As discussed earlier, private goods are typically secondary metabolites that are only accessible by producer cells, including QSI-resistant cells and QS^+ cells (if their signal reception is not inhibited), therefore providing exclusive fitness benefits to the producer cells. In the following three scenarios, we model the 10-day biofilm evolution consisting of QS^- cells, QS^+ cells, and QSI-resistant cells where QS^+ and QSI-resistant cells produce private goods in response to QS activation. Similarly, we consider three QSI strategies: a) targeting signal generation (SE8), b) targeting signal accumulation (SE9), and c) targeting signal reception (SE10).

Compared to SE5, SE6, SE7, coupling between QS-regulated products and bacterial fitness leads to dramatically different evolution results, yet each QSI strategy present distinct risks of the development of resistance. Specifically, targeting signal accumulation results in weak resistance selection while targeting signal reception results in strong selection of QSI-resistance cells. Nevertheless, targeting signal molecule accumulation in the extracellular space continue to show potential as the best approach.

Supplementary Note 4: Simulations *with* QSI Effects and QSI Resistance, where QS Leads to Production of Diffusive Public Goods

As previously shown, the QS-mediated production of diffusive public goods shared by all cells imposes a significant negative impact on the fitness of QS^+ cells. However, under QSI treatment, an important, but often overlooked detail is whether or not QS^+ cells remain actively producing public goods. If not, QS^+ cells incur no metabolic cost and are able to achieve growth rates comparable to QS^- cells. In the following three scenarios, we consider the 10-day biofilm growth consisting of QS^- cells, QS^+ cells, and QSI-resistant cells where QS^+ and QSI-resistant cells produce diffusive public goods in response to QS activation. Similarly, we consider three QSI strategies: a) targeting signal generation (SE11), b) targeting signal accumulation (SE12), c) targeting signal reception (SE13).

In general, QSI therapy works well when the micro-environment at infection sites encouraging QS^{+} and QSI-resistant cells to generate public goods, and the public goods can be effectively shared by QS^{-} cheaters. More precisely, QSI treatment not only shuts down the QS-regulated expression immediately, but also prevents the increase of resistance and QS^{+} genotype ratio (see Supplementary Fig. 8).

Supplementary Note 5: Robustness of Simulation Parameters

We examined the impact of considering different ratios of each strain at the beginning of simulations. More precisely, we use scenario M5 as an example to illustrate that the initial ratio among genotypes does not change the long-term evolution dynamics. Here, we consider four different composition ratios of QS^{+}, QS^{-} and QSI-resistance cells. As shown in Supplementary Fig. 10, when the initial compositions of QSI-resistance cells increases, QSI-resistance cells enter the exponential growth phase earlier; this is due to the fact that QSI-resistance cells begin to massively produce QS-controlled public goods earlier. However, the long-term evolution dynamics is robust and not influenced by this selection of initial composition ratios of the three genotypes of cells (e.g, the QSI-resistance cells have growth advantage compared to other cell types).

We also conduct a robustness analysis of the model parameters to show the range of model parameters that can still capture the desired behavior. Since we calibrate the model parameters to fit the relative changes of the LasR-AHL concentration, the model parameters depend on the relative values. For example, the production rates k_A and k_R in eq (2) and (3) (in the main manuscript) are calibrated to be equal (i.e., $k_A = k_R$) and the binding and unbinding rates are calibrated to be of the same magnitude order. Therefore, we vary the values of these parameters to verify the robustness of the model.

As shown in Supplementary Table 3, the model parameters can accommodate 100X changes with respect to the nominal values we used in the simulations.

Simulator	Environment	Intracellular Modeling	Physicochemical Interaction	Intercellular Interaction
Our Model	Complex 3D	Multiscale stochastic	Yes	Yes
NFSim[12]	Flat 1D	Stochastic simulation	No	No
AgentCell[13]	3D	Stochastic simulation	No	No
RapidCell[14]	2D	Stochastic ODE	No	No
Bsim[15]	Complex 3D	Rule-based	Yes	No

Table 1. Comparison of our proposed model with current bacteria simulators and models.

Parameter	Symbol	Value	Source
AHL maximum production rate	k_A	$2e-3/s$	[4]
$LasR$ maximum production rate	k_R	$2e-3/s$	[4]
AHL constitutive production rate	c_A	$1e-4/s$	[4]
$LasR$ constitutive production rate	c_R	$1e-4/s$	[4]
$LasR-AHL$ half saturation concentration	K_C	$1\mu M$	this study
Small molecule decay rate	k_0,k_3	$1e-4/s$	this study
Small molecule decay rate	k_6,k_7,k_{10}	$1e-4/s$	this study
Binding rate of $LasR$ and AHL	k_1	$1e-1/s$	[4]
Unbinding rate of $LasR-AHL$	k_2	$1e-1/s$	[4]
Formation rate of $LasR-AHL$ complex	k_4	$1e-1/s$	[4]
Deformation rate of $LasR-AHL$ complex	k_5	$1e-1/s$	[4]
QSI_{LasI} half saturation concentration	$K_{QSILasI}$	$5\mu M$	this study
Maximum enzyme reaction rate	k_{cat}	$1e-1/s$	this study
Michaelis constant	K_M	$1e-1$	this study
Binding rate of $LasR$ and QSI_{anlg}	k_8	$2e-1/s$	[4]
Unbinding rate of $LasR-QSI_{anlg}$	k_9	$1e-1/s$	[4]
Cell mass growth rate	k_X	1.4e-4/s	[5]
Half-saturation of nutrition concentration	K_g	$3.7\mu M$	[5]
Maintenance rate	m	$1.5e-7/s$	[5]
EPS maximum production rate	k_{EPS}	$1/s$	this study
DGE maximum production rate	k_{DGE}	$1e-4/s$	this study
Half-saturation of scarce nutrition concentration	$K_{S_{is}}$	$1\mu M$	this study
chelation rate	β	$1.92/s$	[5]
Utilization coefficient of cell mass	U_X	0.6	[5]
Utilization coefficient of digestive enzyme	U_{DGE}	0.6	this study
Utilization coefficient of EPS	U_{EPS}	0.6	this study
Utilization coefficient of $LasR$	U_R	0.6	this study
Utilization coefficient of AHL	U_A	0.6	this study
Biomass and inert density	ρ_X	150	[6]
EPS density	ρ_{EPS}	30	[6]
Diffusion coefficient of extracellular molecules	D_{ex}	$1e-8cm^2/s$	[7]
Boundary layer thickness	T_b	25 μm	this study
Scaling factor for the influential distance	α	10 μm	this study
Saturation factor for regulator complex C	β	1e-3 μM	this study

Table 2. Table of model parameters

Parameter	Symbol	Value	[min, max]	Source
AHL maximum production rate	k_A	$2\times10^{-3}/s$	$[2\times10^{-4}, 2\times10^{-2}]$	[4]
$LasR$ maximum production rate	k_R	$2\times10^{-3}/s$	$[2\times10^{-4}, 2\times10^{-2}]$	[4]
AHL constitutive production rate	c_A	$1\times10^{-4}/s$	$[1\times10^{-5}, 1\times10^{-3}]$	[4]
$LasR$ constitutive production rate	c_R	$1\times10^{-4}/s$	$[1\times10^{-5}, 1\times10^{-3}]$	[4]
$LasR-AHL$ half saturation concentration	K_C	$1\mu M$	$[1\times10^{-7}, 1\times10^{-5}]$	this study
Small molecule decay rate	k_0,k_3	$1\times10^{-4}/s$	$[1\times10^{-5}, 1\times10^{-3}]$	this study
Binding rate of $LasR$ and AHL	k_1	$1\times10^{-1}/s$	$[1\times10^{-2}, 1]$	[4]
Unbinding rate of $LasR-AHL$	k_2	$1\times10^{-1}/s$	$[1\times10^{-2}, 1]$	[4]
Formation rate of $LasR-AHL$ complex	k_4	$1\times10^{-1}/s$	$[1\times10^{-2}, 1]$	[4]
Deformation rate of $LasR-AHL$ complex	k_5	$1\times10^{-1}/s$	$[1\times10^{-2}, 1]$	[4]

Table 3. Robustness of model parameters

Supplementary Video 1: Biofilm Formation in Homogenous Populations of Bacteria

Supplementary Video 2: Biofilm Formation in Heterogeneous Populations of Bacteria

Supplementary Table

Supplementary References

References

1. Hoang, T. T., Sullivan, S. A., Cusick, J. K. & Schweizer, H. P. beta-ketoacyl acyl carrier protein reductase (fabg) activity of the fatty acid biosynthetic pathway is a determining factor of 3-oxo-homoserine lactone acyl chain lengths. *Microbiology* **148**, 3849–3856 (2002).
2. Reimmann, C., Ginet, N. & Michel, L. Genetically programmed autoinducer destruction reduces virulence gene expression and swarming motility in Pseudomonas aeruginosa PAO1. *Microbiology* 923–932 (2002).
3. Smith, K., Bu, Y. & Suga, H. Library screening for synthetic agonists and antagonists of a Pseudomonas aeruginosa autoinducer. *Chemistry & biology* **10**, 563–571 (2003).
4. Melke, P., Sahlin, P., Levchenko, A. & Jönsson, H. A cell-based model for quorum sensing in heterogeneous bacterial colonies. *PLoS computational biology* **6**, e1000819 (2010).
5. Eberl, H. & Collinson, S. A modeling and simulation study of siderophore mediated antagonism in dual-species biofilms. *Theoretical Biology and Medical Modelling* **6**, 30 (2009).
6. Lardon, L. A. *et al.* idynomics: next-generation individual-based modelling of biofilms. *Environmental microbiology* **13**, 2416–2434 (2011).
7. Lawrence, J., Wolfaardt, G. & Korber, D. Determination of diffusion coefficients in biofilms by confocal laser microscopy. *Applied and environmental microbiology* **60**, 1166–1173 (1994).
